# Dental calculus evidence of Taï Forest Chimpanzee plant consumption and life history transitions

**DOI:** 10.1038/srep15161

**Published:** 2015-10-19

**Authors:** Robert C. Power, Domingo C. Salazar-García, Roman M. Wittig, Martin Freiberg, Amanda G. Henry

**Affiliations:** 1 Max Planck Research Group on Plant Foods in Hominin Dietary Ecology, Max Planck Institute for Evolutionary. Anthropology, Deutscher Platz 6, 04103 Leipzig, Germany; 2 Department of Archaeology, University of Cape Town, Cape Town, South Africa; 3 Departament de Prehistòria y Arqueologia, Universitat de València, València, Spain; 4 Department of Human Evolution, Max Planck Institute for Evolutionary Anthropology, Deutscher Platz 6, 04103, Leipzig, Germany; 5 Department of Primatology, Max Planck Institute for Evolutionary Anthropology, Deutscher Platz 6, 04103 Leipzig, Germany; 6 Taï Chimpanzee Project, Centre Suisse de Recherches Scientifiques, Abidjan, Cote d’Ivoire; 7 Institute of Botany, University of Leipzig, 04103 Leipzig, Germany

## Abstract

Dental calculus (calcified dental plaque) is a source of multiple types of data on life history. Recent research has targeted the plant microremains preserved in this mineralised deposit as a source of dietary and health information for recent and past populations. However, it is unclear to what extent we can interpret behaviour from microremains. Few studies to date have directly compared the microremain record from dental calculus to dietary records, and none with long-term observation dietary records, thus limiting how we can interpret diet, food acquisition and behaviour. Here we present a high-resolution analysis of calculus microremains from wild chimpanzees (*Pan troglodytes verus*) of Taï National Park, Côte d’Ivoire. We test microremain assemblages against more than two decades of field behavioural observations to establish the ability of calculus to capture the composition of diet. Our results show that some microremain classes accumulate as long-lived dietary markers. Phytolith abundance in calculus can reflect the proportions of plants in the diet, yet this pattern is not true for starches. We also report microremains can record information about other dietary behaviours, such as the age of weaning and learned food processing techniques like nut-cracking.

Understanding feeding ecology is crucial for recognising the evolutionary pressures that shaped the great apes and humans. It is long recognized that factors such as dietary specialization, tool-assisted food acquisition and the weaning age of infants are important in great apes and humans, and differ significantly among species^1^,^2^,^3^,^4^.

However, many approaches to dietary reconstruction leave unanswered specific questions on diet and related life history events, especially for fossil specimens. There is a need for new methods to reconstruct food acquisition from populations that can avoid some of the shortfalls of other techniques like direct observation, stable isotope analysis and microwear studies^5^,^6^. In some contexts direct observation is simply not possible, for example with extinct great apes and human groups. Stable isotope analysis and dental microwear studies fail to provide total dietary data, and instead only give a picture of broad dietary patterns such as consumption of particular plant categories or mechanical properties of diet^7^,^8^. Furthermore, even where direct observational data on food acquisition is available, data collection is frequently constrained because observation is only feasible over a short window of the lifetime of an individual that may live up to several decades.

Dental calculus sampled from living or dead individuals is rapidly gaining recognition as an invaluable material for the reconstruction of life history. Since Armitage^9^ first recognized plant remains from the teeth of ungulates, studies have reported starch grains, phytoliths, pollen grains, diatoms, mineral particles, proteins and DNA from diverse human and animal populations^10^,^11^,^12^,^13^,^14^,^15^. Using dental calculus from present day forager-horticulturalists, Leonard and colleagues^16^ showed for the first time that recovered microremains also occur in consumed foods, verifying the link between microremains in calculus and diet. As demand grows for dietary history data, analysis of phytoliths and starches in dental calculus is been increasingly used to reconstruct dietary ecology and ecological niches^13^,^17^,^18^,^19^,^20^,^21^,^22^,^23^.

Despite the promise of calculus dietary studies, they are hindered by the lack of research that cross-validates the dietary material recovered in calculus with the organism’s actual feeding ecology and life history. Until recently, our understanding of what the plant matter preserved in calculus precisely represents has been speculative. The initial effort to characterise the microremain record by Leonard and colleagues^16^ reported that calculus captured only a limited proportion of dietary breadth. In this study many vegetable foods lacked phytoliths and starches, and cooking may have significantly reduced starch abundance even if present. Dietary patterns were established through interviewing and short-term camp stays by Leonard, and though the recovered microremains corresponded to the average diet of the population, the dietary records lacked insight into the long term life history of individuals. Without dietary records that cross intra- and inter-annual cycles, our knowledge of the nature of the calculus record and its potential for archaeological studies is incomplete. Furthermore, it is unclear if the calculus dietary record has input from non-dietary sources (e.g. preparation of plant-based tools, oral hygiene and consumption of stomach contents^24^,^25^,^26^), with bias from diagenetic and taphonomic factors rendering it ultimately purely stochastic.

In our study, we compare the plant microremains from the calculus of the chimpanzees of the Taï Forest to 22 years of group averaged (comprising of 128 chimpanzees) dietary observation data in order to validate the calculus record and explore its potential as a source of information on life histories. For this purpose, the study of chimpanzees provides several strengths as a model. First, the chimpanzee mouth is analogous in that chimpanzees often accumulate large deposits of calculus unlike some mammals. Secondly, chimpanzees produce salivary amylase unlike some primates^27^, although it is less abundant than in humans^28^. Thirdly, Taï chimpanzees have a broad diet that includes nearly all food classes (e.g. fruits, piths, leaves, mammals, birds, invertebrates and honey) and is thus relevant to understanding hominin evolution in the African tropics and dietary ecology of hunter-gatherers living in other tropical regions.

We sampled calculus from 24 individual chimpanzees using established methods^18^,^29^, and built a random forest model in R^30^ to allow us to identify the microremains based on multivariate comparison to reference material^31^,^32^,^33^,^34^,^35^ (see detailed methods below). We predict that if microremains reflect diet, they are accumulative in calculus and should increase with age of the individual. Chimpanzee sex might also influence the abundance of microremains, since male and female chimpanzee are known to vary in their time allocation to different food resources^2^,^36^,^37^,^38^. We also anticipate that the proportions of microremains from each plant will be determined by the frequency with which that plant is consumed and how abundant the microremains are in the plant tissue. Although we knew the taxonomic identity of the reference plants at the level of species, an important amount of dietary observation data was present only at the genus level. Therefore, we performed our analyses at the genus level in order to have a higher chance of capturing long term dietary averages for the group, and refer only to genera in the text. Except where otherwise noted, our analyses were done at the group level observational data, since the records for individual chimpanzees were not complete enough to provide a detailed overview of life history. We found the phytoliths in dental calculus to be an approximate record of diet, and furthermore that microremains can reflect important behaviour like nut-cracking and episodes of Taï Chimpanzee life history such as the age of weaning. The implications of our findings from this chimpanzee group are significant not only for diet but also for studying hominin major life history events and culture.

## Results

### Identification of the microremain assemblages

**Figure 1:**
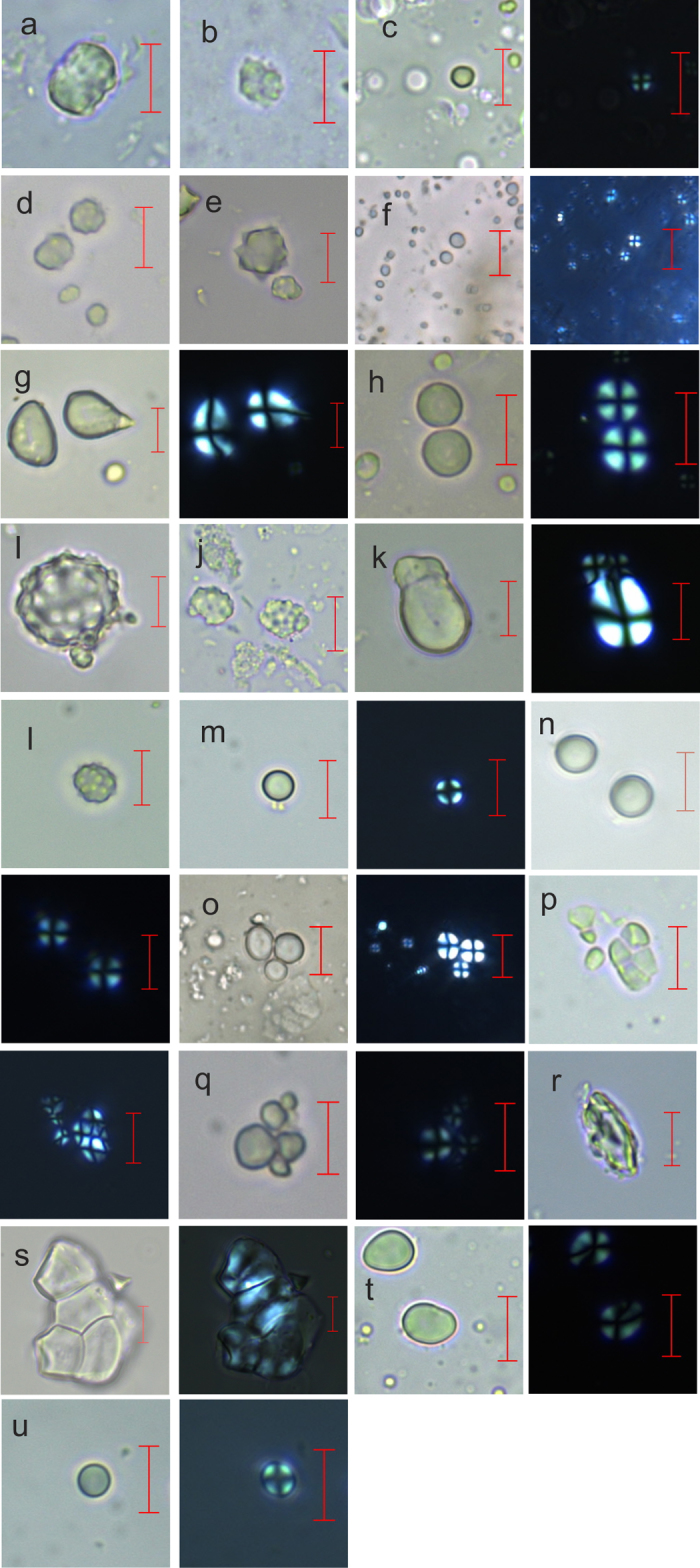
Starch and phytolith morphotypes used in the identification model. Each scale bar represents 10 μm. (**a**) *Aframomum sceptrum* seed phytolith, (**b**) *Aframomum excarpum* leaf phytolith, (**c**) *Aframomum excarpum* seed starch under normal (left) and cross polarized light (right), (**d**) *Laccosperma opacum* pith phytolith, (**e**) *Laccosperma secondiflorum* seed phytolith, (**f**) *Calpocalyx sp.* fruit starch under normal (left) and cross polarized light (right), (**g**) *Cola nitida* seed starch under normal (left) and cross polarized light (right), (**h**) *Coula edulis* seed starch under normal (left) and cross polarized light (right), (**i**) *Elaeis guineensis* fruit phytolith, (**j**) *Elaeis guineensis* leaf phytolith, (**k**) *Gilbertiodendron splendidum* seed starch under normal (left) and Cross polarized light (right), (**l**) *Eremospatha macrocarpa* pith phytolith, (**m**) *Eremospatha macrocarpa* pith starch under normal (left) and cross polarized light (right), (**n**) *Napoleona leonensis* seed starch under normal (upper right) and cross polarized light (lower left), (**o**) *Panda olesosa* seed starch, (**p**) *Piper guineense* seed starch under normal (upper right) and cross polarized light (lower left), (**q**) *Sacoglottis gabonensis* fruit starch under normal (left) and cross polarized light (right), (**r**) *Sarcophrynium prionogonium* fruit phytolith, (**s**) *Sarcophrynium prionogonium* fruit starch under normal (left) and cross polarized light (right), (**t**) *Treculia africana* seed starch under normal (left) and cross polarized light (right), (**u**) *Xylia evansii* seed starch.

We were able to examine 91 of the 157 genera (113 of 230 species) of plants that the 128 Taï chimpanzees consumed during the observation period. Of these plants, only a small subset produced sufficiently diagnostic microremains to use for identification (thirteen starch- and five phytolith-producing genera, Table 1; Fig. 1). For each microremain-producing plant genus, we collected data from 50 microremains, to provide a range of measurements within each genus. We collected 9 types of measurements for phytoliths and 11 for starches from 900 microremains (Supplementary Data 1 and 2). By using a subset of the reference collection to test the model, we assessed the success rate of identification of each genus with the model. Some genera were reliably identified, and others were more difficult to identify. For example, *Sarcophrynium* phytolith*s* were identified successfully 94% of the time while *Panda* starch was only identified 22% of the time. Generally, phytoliths were identified more reliably (Supplementary Table 1, 2). Using this random forest model we were able to proceed with identification of microremains recovered from the calculus.

**Figure 2:**
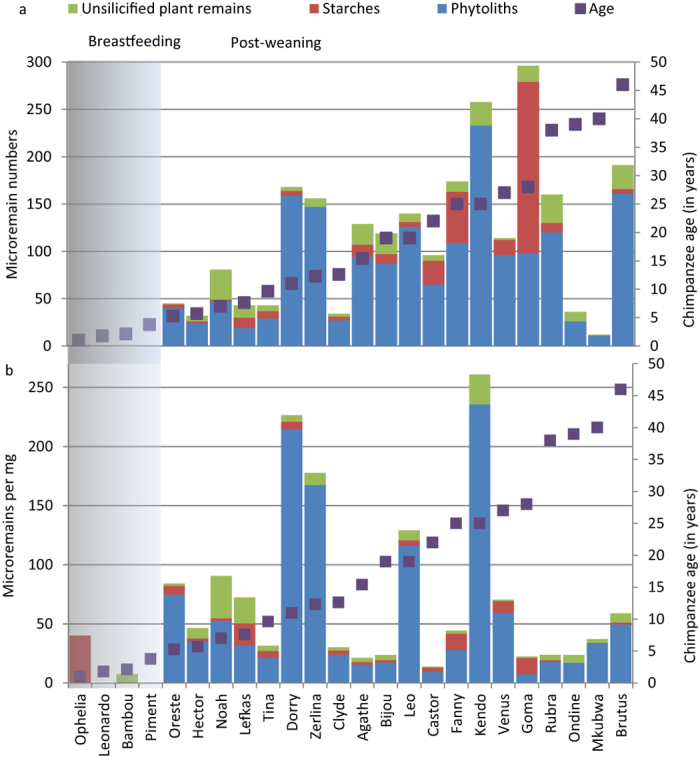
Unsilicified microremains, starches (definite and possible) and phytoliths recovered in Taï Chimpanzee dental calculus with chimpanzee age at death (in years) and approximate age of the cessation of weaning highlighted. (**a**) Total counts and (**b**) counts per milligram of calculus. The number of microremains per mg in Ophelia was affected by an unusually small amount of calculus in the sample.

**Figure 3:**
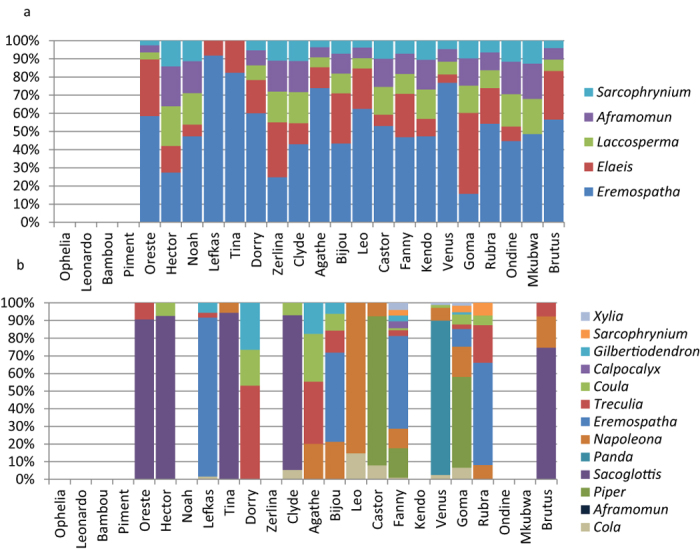
Microremain assemblages recovered in calculus. (**a**) Bar chart of the composition of the phytolith assemblage recovered from calculus. (**b**) Bar chart of the composition of the starch assemblage recovered from calculus. The individuals are ordered by age from youngest to oldest.

Of the 24 chimpanzee calculus samples, we found starches in 17 of the samples, and phytoliths in 20 (Figs 2 and 3; Supplementary Table 3; Supplementary Data 3). We also found unidentified phytoliths, unsilicified plant fragments, diatoms, pollen and insects, but these were not identified to taxon (Supplementary Data 3). In ambiguous cases microremains were classified as possible starches and specifically stated, but were not used for statistical genera identification. Most definite starches and phytoliths that were free from damage (234 starches and 1035 phytoliths) were identified to genus using the random forest model, which assigned each unknown microremain to a genus and provided a certainty score that indicated the confidence with which that assignment was made. A microremain was considered to be damaged if it showed pitting, ruptured surfaces or other major irregularities. The highest certainty score for each individual microremain depended heavily on each genus identification rate (as described above), but generally ranged between 0.25 and 0.95.

### Assessment of biases in our data

First of all, it was important to ascertain if the treatment of the skeletal material to prevent the spread of disease (including one year of burial, and various chemical treatments) had impacted microremain preservation in the calculus. After 2004, chorine and formalin was used to clean skeletal material. Boiling may have been used on some skeletons to clean them and remove Ebola pathogens between the Autumn of 1994 and the Spring of 1996 (Supplementary Table 3; Supplementary Fig. 1). To test if the three types of treatments significantly influenced starch preservation we used a Kruskal-Wallis test on starch per mg on samples from each period (H = 3.7633, df = 2, p-value = 0.1523). We included microremains classified as possible starches in the starch per mg count. Due to the small sample size, we calculated a Kruskal-Wallis p-value based on 999 random permutations. This indicated no differences between the three groups (Permutation H = 7.1215, df = 2, p = 0.159). Previous studies of other organic material (bone collagen) in the Taï skeletal collection have indicated no significant post-mortem alteration^36^,^39^. While collagen does not necessarily behave in the same manner as plant microremains, it is likely that the comparable hydroxyapatite mineral matrices of bone and calculus have a similar protective effect on the organic materials trapped within them^40^.

**Figure 4:**
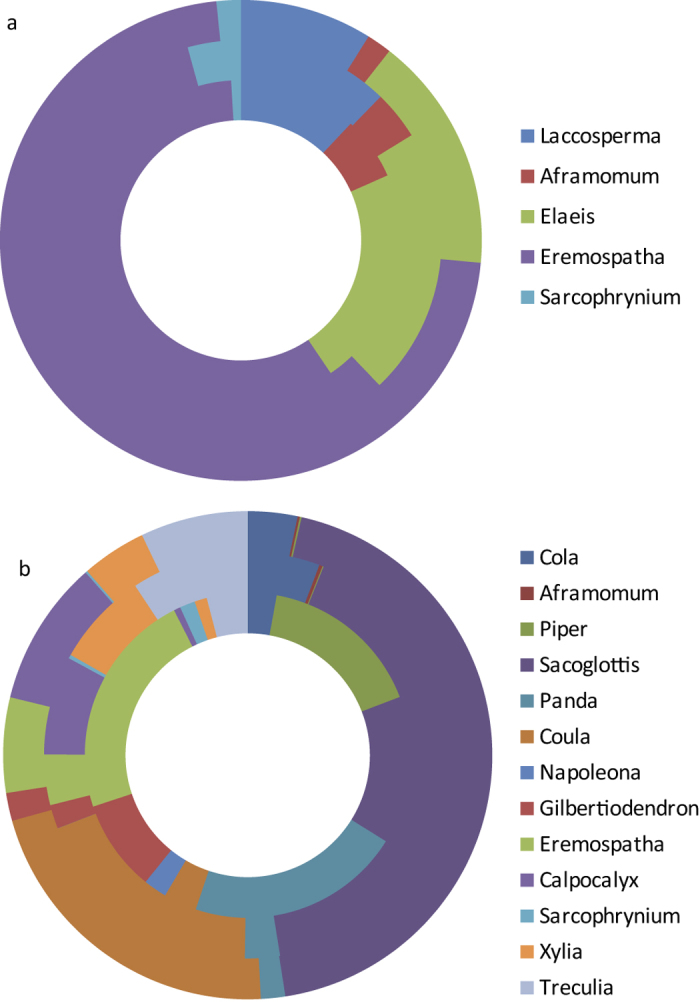
Microremain assemblages recovered in calculus. Microremain counts are normalised by dividing counts by the percent content of dry plant weight of starches and phytoliths among different genera. (**a**) Phytolith counts compared with feeding records. Outermost ring = proportions of minutes spent consuming each genus averaged across the feeding records of sampled 24 sampled chimpanzees, middle ring = proportions of minutes spent consuming each genus averaged across the feeding records of all 128 chimpanzees, innermost ring = phytolith counts from the sampled 24 chimpanzees. (**b**) Starch counts compared with feeding records: outermost ring = proportions of minutes spent consuming each genus averaged across the feeding records of sampled 24 chimpanzees, middle ring = proportions of minutes spent consuming each genus averaged across the feeding records of all 128 chimpanzees, innermost ring = starch counts.

Before comparing the calculus results to the observational records, we wanted to see if there was excessive variation in plant representation among the calculus samples. Phytoliths from four of the five phytolith-producing genera were found on most, but not all, of the calculus samples, suggesting that there is not much variability among these calculus samples. Some genera are found in each sample (*Eremospatha* and *Elaeis*) while others, like *Sarcophrynium,* were rare. However, the starch record varies significantly among individuals, with most of the thirteen starch-producing genera seldom found. This probably reflects the far lower numbers of starches compared with phytoliths. Several genera dominate the starch record, namely *Gilbertiodendron*, *Coula*, *Eremospatha*, *Treculia* and *Cola* (Fig. 3; Supplementary Table 4). Most microremains were isolated, but three calculus samples had four starch aggregates from *Piper*; each starch in the aggregate was counted as an individual starch granule as counting each was not possible and thus constitutes a large proportion of the total number of the recovered starches. This potentially biases the starch assemblage’s dietary representativeness (Figs 3 and 4). In sum, it seems that there is not much variation in the phytolith record of our chimpanzee samples, but the starch record is less homogeneous.

Another potential source of bias comes from the differential preservation of microremains relating to their inherent properties, like size and shape. We noted that our results were biased towards foods with larger sized microremains. *Elaeis* phytoliths and *Cola* starches, the largest microremains in the study (Figs 1 and 4), are disproportionately frequent across the assemblages even after controlling for the high concentration of microremains within these genera. They are frequently found, but are not dominant foods (Supplementary Fig. 2).

### Microremain accumulation, chimpanzee age and sex

We predicted that number of microremains should increase with age, and might vary by sex. We tested this using a negative binomial regression, with microremain count as the response, and age and sex as predictors, weighing each observation by the weight of the calculus sample (see detailed methods below). We ran separate tests for phytoliths, starches and unsilicified remains. For phytoliths, the full model of age and sex significantly influenced the count of phytoliths (x^2^ = 11.794, df = 2, P = 0.0003), and the effect of age was also significant by itself (x^2^ = 12.753, df = 1, P = 0.0004) (Supplementary Table 5). Older chimpanzees generally have a higher abundance of phytoliths. However, sex by itself did not explain the abundance of phytoliths we found (x^2^ = 0.028, df = 1, P = 0.866). For unsilicified microremains, age and sex as the full model significantly influenced the microremain count (x^2^ = 10.067, df = 1, P = 0.015), the effect of age alone was also significant (x^2^ = 9.202, df = 1, P = 0.0015), but not sex by itself (x^2^ = 0.59, df = 1, P = 0.806). Starch abundance was significantly determined by age and sex together (x^2^ = 23.994, df = 2, p = 6.1622e–06). Older chimpanzees generally have a higher abundance of starches (x^2^ = 3.559, df = 1, p = 0.0592). Unlike with phytoliths and unsilicified remains, sex strongly influenced the abundance of starch (x^2^ = 17.301, df = 1, p = 3.1897e–05), with female chimpanzees having more starches (Supplementary Table 5).

### Microremain dietary picture and observational feeding records

We predicted that more frequently consumed plants should be highly represented in the chimpanzee calculus. To test this, we used an observational random effect Poisson model (Supplementary Text 5). The count of identified classes of microremains (phytoliths and starches) belonging to a particular genus was our response variable, and the fixed predictors were: (a) minutes spent consuming each genus, and (b) chimpanzee age in months. Sex was included as a control predictor, and both calculus sample weight and successful identification rate of each genus were included as weights. We used counts of each genus predicted to be present with the total minutes spent consuming each genus. The chimpanzee individual was included as a random slope term, while year of death, tooth and food type were treated as random intercept terms (see methods below for more details).

**Figure 5:**
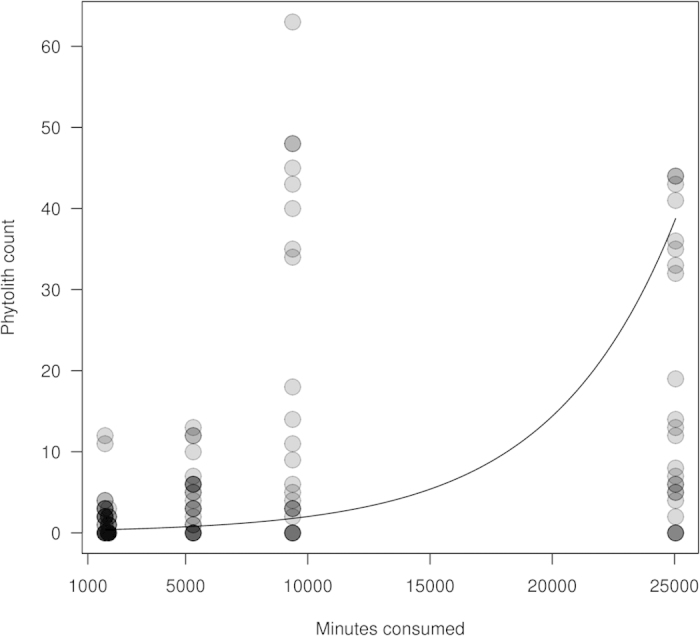
Plot of Mixed Poisson regression model. The number of phytoliths from a genus increased as the minutes spent consuming this plant resource increased. Darker circles reflect overlapping values.

When comparing the genera proportions present in the diet (calculated as the number of minutes spent foraging on a genus) with the recovered phytolith assemblages, we found a clear relationship. The amount of minutes spent consuming a given plant genus influences its phytolith count in the calculus assemblage (Figs 4 and 5), even when accounting for the effects of sex, the tooth we sampled, variation in phytolith production between different plants, and the year the individual died. More specifically, an increased reliance on a genus leads to an increase in its representation in calculus (x^2^ = 4.048, df = 1, P = 0.045; Supplementary Table 5). The age of the chimpanzees did not influence how well it matches group diet (x^2^ = 0.356, df = 1, P = 0.55 Supplementary Table 5).

In contrast to phytoliths, there was no significant effect of consumption time on starch numbers (x^2^ = 1.95, df = 2, P = 0.376). The number of minutes this group spent eating a specific genus of starchy foods does not influence its frequency in dental calculus. Yet there is an element of uncertainty because starches vary more among individuals than do phytoliths, as described above, and do not seem to be as good a record of dietary behaviour. Figures 3 and 4 show the discrepancy between the consistency of starch and phytolith results clearly. These results may be a product of post-mortem diagenesis that impacted our chimpanzee samples, including burial to deflesh the remains (Supplementary Table 3). Because these processes are likely unique to our sample, we cannot assume that the same confounding factors will always be present in calculus from other groups.

### Weaning and other behavioural signatures in calculus

**Figure 6:**
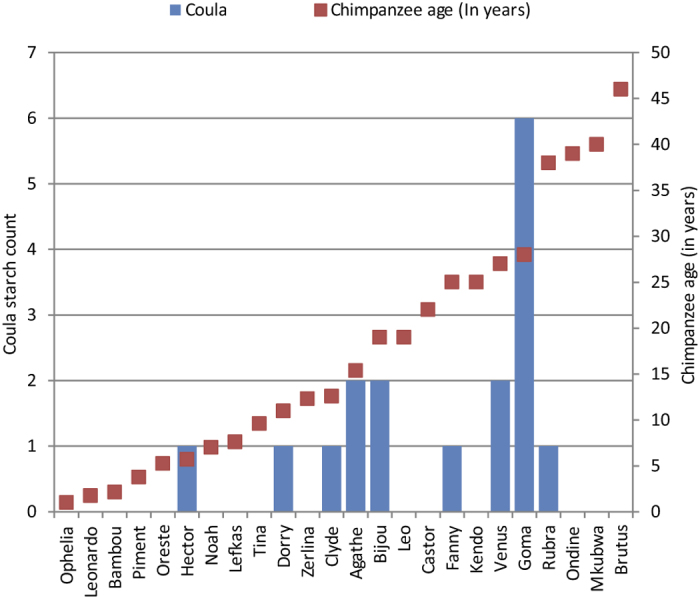
The occurrence of *Coula* nut starches with chimpanzee age at death (in years). *Coula* nut consumption requires nut cracking and its presence implies nut cracking and tool use or food sharing. The individuals are ordered by age from youngest to oldest.

The microremains in the Taï calculus record other aspects of their behaviour. First, microremains were strikingly rare in samples from individuals less than 5.3 years old (Figs 2 and 6; Table 2). The calculus deposits were sparse on these individuals, but despite the small volume of calculus, it was notable that only a single starch and an unsilicified plant fragment were found in these samples. Chimpanzees more than 5.3 years old typically show high numbers of microremains, regardless of the size of the calculus deposit.

Second, the exact plants that were recovered in the calculus provide an interesting view of an important learned behaviour. In our sample, many calculus samples had starches from the *Coula* nut, which is mainly consumed once chimpanzees learn to crack open these nuts. *Coula* nut starches were found in samples from individuals across all age ranges (except those under 5.7 years) (Fig. 6). Although common, *Coula* nut appears to be underrepresented in our sample. It was found only in nine calculus samples, despite this plant being a major food source, comprising of 4.7% of the total Taï diet.

## Discussion

Much of the chimpanzee calculus is relatively rich in plant microremains compared with what has been reported in previous studies of human calculus^11^,^16^,^18^. This is not entirely unexpected for several reasons. First, our samples are modern, and post-mortem microremain diagenesis is therefore less acute than in ancient remains. Secondly, Taï Chimpanzee diet is plant-dominated and voluminous (Supplementary Fig. 1). Thirdly, and in contrast to humans, chimpanzees consume a large amount of phytolith-rich material. This richness in microremains is largely confined to phytoliths. Starch abundance falls within ranges observed elsewhere^12^,^16^,^18^.

It is evident that starches are underrepresented, and in some samples are even totally absent. In addition, phytoliths present a far more uniform picture of diet between different chimpanzees. This may be due to diagenesis occurring during the preparation of the skeletons for the osteology collection that preferentially alters or removes starches from the calculus record. It is known that all skeletons were buried for short periods of time during the defleshing process (Supplementary Table 3). These processes may preferentially alter or remove starches from the calculus record that are not sufficiently trapped and sealed, while leaving the phytoliths relatively unaltered. Fortunately, our Kruskal-Wallis test indicates cleaning processes have not influenced starch numbers.

Additionally, we found that the microremain record was likely biased by the differential survivability of microremains from different plants. The plants with the largest starches and phytoliths were overrepresented in our sample, possibly due to the larger surface area. This ties in with research that shows that phytolith and starch morphology and surface area is linked to long term stability^41^,^42^. Larger blocky microremains may be preferentially preserved.

Overall, our results verify that the calculus record can be accumulative by showing that older individuals present more microremains. Sex may be a factor to take into consideration, and seems to influence the accumulation of starches, but not phytoliths or unsilicified remains. It may reflect higher consumption of starches by female chimpanzees, or sex differences in amylase production or calculus formation, as has been suggested for humans^43^. We do not currently have the ability to distinguish among these possibilities. The increase in microremains with age and possibly sex implies that microremain accumulation is bound up in aspects of diet that regulate calculus formation. Thus, microremain presence and proportions are likely effected or confounded by all the factors that influence dental plaque and calculus (i.e. intake of protein, smoking, polysilicic acid and silica)^44^,^45^,^46^. Calculus clearly can approach a long-term dietary signal, although the timespan involved is not yet clarified.

Our results strongly suggest that care must be taken when interpreting the microremains record preserved in dental calculus, particularly the starch grain record. However, our results also indicate that microremains in calculus can be used to recover important information about diet, behaviour and life history. For example, we observed a lack of microremains from deciduous teeth of chimpanzees less than 5.3 years old. This pattern matches what is generally reported for age of weaning by using other measures. Much information on the age of chimpanzee weaning is estimated from inter-birth interval (IBI)^39^. IBI estimates of weaning ages vary from 4.5 years at Gombe to 5.75 years at Taï^47^, to 6 years at Mahale^48^. Yet IBI is an indirect measure as it includes more than simply suckling duration. Isotopic based data indicates weaning at Taï commences at 2 years and carries on till 3–4.5 years, varying by factors such as sex of the offspring.

Thus, microremain assemblages could indicate a rapid accumulation of microremains as solid food dominates the diet (Fig. 2). If we combine this trend together with the verification of the accumulative nature of the microremain assemblages, we can conclude calculus reflects information on the weaning transition that may be useful for studying unhabituated populations.

Furthermore, though the starch dietary record appears more stochastic than that of phytoliths, starches can still provide useful information about behaviour. Many of our starches come from the *Coula* nut (Fig. 6). Among chimpanzees, *Coula* consumption requires a learned behaviour: nut cracking with a hammer and anvil. This behaviour is restricted to a limited area of the chimpanzee range in West Africa^1^. The presence of *Coula* starches (Figs 3 and 6) shows calculus can reveal nut-cracking behaviour in a group. The fact that tool-use in a group is discernible is relevant for dental calculus studies in both primatology and hominin evolution. The use of *Coula* nut is influenced by age and sex differences in nut cracking^2^,^49^, and, as expected, *Coula* starches are absent in youngest chimpanzees who are not yet weaned. Even after weaning infant nut consumption is low and is derived from nuts cracked by the mother as it takes years to learn how to crack nuts^2^. Beyond this, we do not have enough calculus samples to examine if there are sex or age differences in the calculus record of nut cracking.

In summary, the study verifies the relevance of dental calculus for investigations on diet, food acquisition behaviour and life history. It is the first to link dental calculus with foods that entered the oral cavity in quantified abundances. The data also provide valuable information on the commencement of plant food consumption in wild chimpanzees, and confirm the consumption of solid foodstuffs from at least 5.3 years in life. Our study suggests that calculus analysis provides a rich but wavering insight into complex dietary structure, and that phytoliths, when present in calculus and in diet, may provide a more reliable record of chimpanzee diet than starches.

## Materials and Methods

### Taï Forest reference material

A reference collection with 91 genera (113 species) of the most frequently consumed chimpanzee plant foods in the Taï Forest was collected and examined for phytoliths and starches (Supplementary Table 6). Phytoliths and starches were isolated from reference plants using conventional approaches^50^. We selected thirteen starch- and seven phytolith-producing genera from the 91 we analysed for the identification model (Supplementary Text 3).

### Calculus sampling

The calculus samples used for our analysis come from permanent and deciduous molars of 24 chimpanzee individuals from the Taï Chimpanzee Osteology Collection at Max Planck Institute for Evolutionary Anthropology (MPI-EVA) with varied life histories (Supplementary Text 1, 2; Supplementary Table 1). We selected only molars to standardise the sampling, and chose teeth that were encrusted with a prominent band of supragingival calculus (calculus present above the original gumline) on the enamel crown. Deposits of supragingival calculus were present on all individuals ≥1 year old. Subgingival calculus was also present but was not sampled since it occurs below the former gums and it is unclear if it preserves food remains. Calculus on the teeth was documented with photography before sampling, and the colour noted with how each skeleton was treated before our sampling (Supplementary Table 1). Packing material was sampled as a control. An unidentified adhesive used in the curation of some specimens was removed before sampling. A dental scalar was then used to remove small areas of calculus. The amount of calculus sampled had no relationship with the amount of calculus present on the teeth except in the youngest chimpanzees (<5.3 years), where calculus was rare and almost entirely collected. We sampled in clean conditions in a laminar flow cabinet at positive-pressure at the MPI-EVA. We then weighed each of the samples and transferred them to microcentrifuge tubes. After sampling, the teeth and surviving calculus were photographed.

Some studies have highlighted the risks of laboratory contamination from modern plant microremains^51^,^52^,^53^. To address the possibility of contamination, we conducted a regime of weekly laboratory cleaning to remove contamination. All work surfaces were wiped with hot water, washed with starch-free soap and wiped with 5% sodium hydroxide (NaOH). We additionally performed wipe tests before and after weekly cleaning to quantify starch contamination and assess contaminating types. Wipe tests retrieved settled particles of the surface area (74 × 43 cm^2^) of the laboratory positive-pressure laminar flow hood used for mounting. Results of these intensive contamination control tests are in Supplementary Data 4.

### Optical microscopy

Optical microscopy was performed at the Plant Foods in Hominin Dietary Ecology laboratory in the MPI-EVA (for reference collection microscopy see Supplementary Text 3 and Supplementary Tables 4, 7). We added 150 μl of 10% hydrochloric acid to the calculus samples for one to three hours. The samples were then centrifuged at 1691 × g (Heraeus MEGAFUGE 16 with a microcentrifuge rotor) for 10 minutes and then about 100 μl of supernatant was decanted and replaced with distilled water. This was repeated three times to remove the hydrochloric acid. After the second decanting, they were refilled with a 25% glycerine solution. The samples were then ground in the solution in the 1.5 ml Eppendorf microcentrifuge tube to reduce sample loss due to static electricity. The samples then were centrifuged again at the same speed, and about 1 ml of supernatant was decanted. We mounted 20 μl per slide on as many slides as needed in order to examine the entire sample. Microscopy was used as in conventional phytolith and starch studies^12^,^54^. We examined each slide under brightfield and cross-polarized light on a Zeiss Axioscope microscope at 400× magnification. We photographed each microremain and described each with the international microremain nomenclature including the International Code of Phytolith Nomenclature^55^. In some cases starch aggregates were identified in calculus. In this case, each component granule of each aggregate was counted as an individual starch.

### Microremain identification

We identified microremains with a reference collection using multivariate analysis with a random forest algorithm. We collected five general microremain measurements, four specific to phytoliths and six specific to starches from a total of 900 reference microremains (Supplementary Table 7; Supplementary Data 1 and 2). With this reference collection we generated an identification certainty score for each microremain. The validity was tested through cross-validation with a subset of reference data (Table 1; Supplementary Table 1, 2, 8, 9; Supplementary Data 5). We identified the microremain as coming from the genus with the highest certainty score (Supplementary Data 5 and 6).

### Behavioural records

The chimpanzees of the Taï Forest have been studied since the commencement of the Taï Chimpanzee Project in 1979^2^. The detailed recorded behaviour of the group included observation of feeding time and food item consumed. The feeding records used in our study span the period between 1992 and 2014 (Supplementary Fig. 1). The database includes 1,165,150 million behavioural observations of about 128 chimpanzees, with a total of 417,628 dietary observations (2,380,202 minutes). However, only roughly 30,000 observations come from chimpanzees available for sampling at the osteology collection. Furthermore, most of these chimpanzees have only sporadic coverage of their life history. Therefore, instead of using dietary records of individual chimpanzees or the collated records of the 24 chimpanzees we sampled, we chose to combine dietary records from all 128 chimpanzees to best represent the average Taï Forest diet.

The feeding record includes the times when a chimpanzee started and finished eating, and the food consumed. We chose only those feeding records where the genus of the plant food eaten was documented, and calculated the total amount of time spent consuming each resource. Behavioural records do not account for variations in the volume of food consumed in given amount of minutes. In addition, although some observations record the specific plant part that was eaten, most do not, so we do not include this information.

### Statistical analysis

To test for the effects of age on microremains we used a negative binomial regression (log link) with a count of each microremain class treated as a response (phytoliths, starches and other unsilicified plant fragments) using a likelihood ratio test in R 3.1.0^56^. We ran the regression using the glm.nb function of R package MASS^57^. The full model included the fixed effects of age and sex (Supplementary Text 5). The mg weight of each calculus sample was used to weigh the model to account for larger samples likely being more representative of overall diet due to the potential of microremains to have a clustered distribution in the calculus matrix. Controlling for weight, heavier samples have less variable microremain counts (Compare Table 2 with Supplementary Table 1, 2). The full model was compared with a null model using an ANOVA. We used likelihood ratio tests comparing the full models with reduced models in which each fixed effect was dropped, one at a time. Model assumptions were met. Collinearity was not an issue (largest Variance Inflation Factor = 1.001) and leverage values, as well as DFBeta values, indicated no obvious highly influential cases.

To explore the relationship between diet and the phytolith microremains found in dental calculus, we tested an Observational random effect Poisson model with likelihood ratio tests. We used counts of each genus predicted to be present with the total minutes spent consuming each genus. For this, we used the glmer function of the R package lme4^58^. If any genus was not predicted to be present in a chimpanzee sample, they were included as a 0 value. Our full model included minutes and chimpanzee age in months as fixed effects, and sex as a control predictor. The model included the weight of each calculus sample and the successful identification rate of each type of genera as model weights, and used microremain content as an offset to factor in significant differences in content between different genera. To prepare the data, we z-transformed the minutes and age variables. The chimpanzee individual was included as a random slope term while year of death, tooth and food type were treated as random intercept terms. An id was assigned to each observation, and this was also included as a random intercept, thus reducing overdispersion to (x^2^ = 13.369, df = 116, dispersion parameter = 0.115) in the phytolith model. To test the significance of the full model it was compared with a null model excluding fixed effects of minutes of observation and age. Variance inflation factors (VIF)^59^ were derived to assess collinearity using the function vif of the R-package car, from a standard linear model minus random effects, as offsets and weights^60^. Variance inflation factors indicated collinearity to not be an issue (largest VIF = 1.02). We tested model stability by excluding each random effect one by one from the data set, running the full model and comparing the results with those from the original model that suggest no highly influential cases.

To explore the relationship between diet and the starch microremains, we could not use the same approach due to high zero inflation present in the starch data. To overcome this we implemented a Mixed effects logistic regression using the same terms, random effects, weighs and offset as the phytolith Poisson model. This required the count data (the response) to be treated as presence and absence data resulting in some loss of data. Variance inflation factors^59^ were derived to access collinearity using the function vif of the R-package car, from a standard linear model minus random effects as well as offsets and weights^60^. Variance inflation factors indicated collinearity to not be an issue (largest VIF = 1.018).

## Additional Information

**How to cite this article**: Power, R. C. *et al.* Dental calculus evidence of Taï Forest Chimpanzee plant consumption and life history transitions. *Sci. Rep.* **5**, 15161; doi: 10.1038/srep15161 (2015).

## Supplementary Material

Supplementary Information

Supplementary Data

## Figures and Tables

**Table 1:** Plant genera selected from reference collection species for use in the predictive identification model, with the microremain content of the dried plant material provided as a percent of dried plant material, and the frequency of observed consumption provided as number of minutes eaten.

Plant category (Genus)	Microremain type	Plant part	% Microremains /Dry Weight	Minutes eaten
*Elaeis*	Phytolith	Fruit and Leaf	4.81	9379
*Eremospatha*	Phytolith	Pith	1.72	25,046
*Laccosperma*	Phytolith	Pith and Seed	2.15	5311
*Aframomum*	Phytolith	Seed and Leaf	2.13	1704
*Sarcophrynium*	Phytolith	Fruit	3.32	1847
*Cola*	Starch	Seed	40	35,778
*Aframomum*	Starch	Seed	54.58	1704
*Piper*	Starch	Seed	39.22	492
*Sacoglottis*	Starch	Fruit	2.46	258,225
*Panda*	Starch	Seed	0.85	17,299
*Coula*	Starch	Seed	31.15	118,095
*Napoleona*	Starch	Seed	20.79	51
*Gilbertiodendron*	Starch	Seed	23.87	11,808
*Eremospatha*	Starch	Pith	2.93	25,046
*Calpocalyx*	Starch	Fruit	9.1	49,074
*Sarcophrynium*	Starch	Fruit	23.83	1847
*Xylia*	Starch	Seed	19.58	46,587
*Treculia*	Starch	Seed	23.87	58,093

We chose to use genus as the taxonomic rank as some dietary records only identify genus.

**Table 2:** All chimpanzee dental calculus samples analysed.

Name	ID	Tooth	Sex	Weight (mg) of calculus sample	Age at death	
					In years	In months
Ophelia	14993	Lower Left DM1	Female	0.025	1	12
Leonardo	13432	Upper DM2	Male	0.329	1.92	23
Bambou	11777	Lower Left DM1	Male	0.135	2.08	25
Piment	11788	Lower Right DM1	Female	0.27	3.58	43
Oreste	14995	Lower Left M1	Male	0.536	5.25	63
Hector	12175	Upper Right M1	Male	0.689	5.67	68
Noah	15011	Lower M1	Male	1.165	7	84
Lefkas	13433	Upper Left M2	Male	0.595	7.58	91
Tina	11790	Lower Right M1	Female	1.36	9.08	109
Dorry	15020	Lower Right M2	Female	0.742	11	132
Zerlina	11792	Lower Left M3	Female	0.878	12.3	144
Clyde	11779	Lower Right M1	Male	1.131	13	156
Agathe	11775	Lower Right M2	Female	6.076	16	192
Leo	15012	Lower Right M3	Male	1.085	19	228
Bijou	11778	Lower Left M2	Female	5.041	19	228
Castor	13439	Lower Left M1	Female	6.982	22	264
Kendo	11781	Lower Left M2	Male	2.895	25	300
Fanny	11780	Lower Left M3	Female	3.915	25	300
Venus	15001	Upper Right M1	Female	1.133	27	324
Goma	15004	Upper Right M3	Male	13.208	28	336
Rubra	15023	Lower Left M2	Female	6.751	38	456
Ondine	11786	Lower Left M1	Female	1.529	39	468
Mkubwa	13435	Lower Right M3	Male	0.324	40	480
Brutus	15029	Upper Left M3	Male	3.246	46	552
