# Supplementary material for: Dental calculus evidence of Taï Forest Chimpanzee plant consumption and life history transitions
Source: Sci Rep. 2015 Oct 19;5:15161. doi: 10.1038/srep15161 (PMC4611876; doi:10.1038/srep15161)
Supplement: Supplementary Information [file srep15161-s1.pdf]

Supplementary Information for: “Dental calculus evidence of Taï Forest Chimpanzee plant consumption and life history transitions”

Robert C Power[1]*, Domingo C Salazar-García[2,3,4], Roman M Wittig[5,6], Martin Freiberg[7], Amanda G Henry[1]

[1]Max Planck Research Group on Plant Foods in Hominin Dietary Ecology, Max Planck Institute for Evolutionary Anthropology, Deutscher Platz 6, 04103 Leipzig, Germany.

[2]Department of Archaeology, University of Cape Town, Cape Town, South Africa.

[3]Departament de Prehistòria y Arqueologia, Universitat de València, València, Spain.

[4]Department of Human Evolution, Max-Planck Institute for Evolutionary Anthropology, Deutscher Platz 6, 04103 Leipzig, Germany.

[5]Department of Primatology, Max Planck Institute for Evolutionary Anthropology, Deutscher Platz 6 04103 Leipzig, Germany.

[6]Centre Suisse de Recherches Scientifiques, Abidjan, Cote d'Ivoire

[7]Institute of Botany, University of Leipzig, 04103 Leipzig, Germany.

*Corresponding author

+49 (0)341 3550 789 / +49 (0)15237044289

robert_power@eva.mpg.de

Supplementary Texts

1. Study population

The chimpanzee calculus samples derive from the Taï Chimpanzee osteology collection of 77 chimpanzees curated at the Max Planck Institute for Evolutionary Anthropology (MPI-EVA) in Leipzig, Germany. The remains were collected with as many details as possible on sex, age and cause of death. All Taï Forest material and data collected complied with the requirements and guidelines of the Ministère de l’Enseignement Supérieure et de la Recherche Scientifique of Côte d’Ivoire, and adhered to its legal requirements. When possible we sampled chimpanzees who had known life histories, and ideally with comprehensive dietary records. Much of the observational data relates to chimpanzees that are not part of this osteology collection. Dietary records vary from thousands of observations over a decade to a limited number over the course of a single day. After death, these individuals were interred for defleshing and then later exhumed. Some of the skeletal material was cleaned using strong disinfectants before storage to minimize the risk of disease transmission.

It has been noted that chimpanzees produce less salivary α-amylase than humans, especially humans from agricultural societies that consume high levels of starch[1]. Thus starch entering the chimpanzee mouth may be less readily hydrolysed than in human groups, which may make it more likely for starches to enter and preserve in chimpanzee dental calculus than in human

dental calculus. However if this patterns occurs in our samples it is unclear and it cannot testable with our data.

2. Collection of calculus samples

Occasionally, chimpanzee calculus showed substantial flecks of dark material that did not resemble calculus and appeared to be sediment contamination. Chimpanzee samples where sediment contamination was suspected were omitted. All chimpanzee remains sampled are curated at the Max Planck Institute for Evolutionary Anthropology in Leipzig, Germany. Samples from two chimpanzees (Vanessa and 13438) were omitted from analysis because their age at death was not recorded, though we did count microremains recovered from these individuals in the Supplementary Data 3. A sample from a further chimpanzee (Loukoum) was omitted due to surface adherents on the calculus. The calculus we chose for the final complete analysis came from molars of 24 individuals (12 male and 12 female) ranging in age from between 12 and 552 months (1 and 46 years) old (Table 1; Supplementary Table 3).

3. Taï Forest plant reference collection

A microremain reference collection with 119 plant species was built using the most frequently consumed chimpanzee plant foods in the Taï forest (Supplementary Table 7). Taï chimpanzees consume a particularly diverse range of foods. We collected plant parts that were documented as a specific component of the diet (fruits, seeds, piths, leaves, stems, bark, flowers, and roots.) We also include fungal fruiting bodies known to be consumed. Effort was made to include other rainforest edible plants not recorded as chimpanzee foods. Although our reference collection is not exhaustive, it incorporates the most important plants foods of the Taï chimps, achieving coverage of 89 % of the total dietary observations. Plants collected in the Taï Forest were immediately preserved onsite either by freezing or by drying in 15 or 50 ml centrifuge tubes with silica gel (Roth - T858.1 and P077.1, Karlsruhe, Germany). Additionally, we collected some plant material from the University of Leipzig Botanical Garden (marked as fresh in Supp. Table 7) and analysed this material fresh for starch or dried for phytoliths. We did not make a reference collection for unsilicified plant microremains as these microremains are unlikely to be undiagnostic.

Starch was analysed by directly mounting finely sliced dry plant material on slides with approximately 10 µl of distilled water and 10 µl of a 25 % glycerol solution. Starches were observed at 200-640 x magnification using a Zeiss Axioscope. Phytoliths were isolated from plant material by dissolving weighed dried plant material in ≥65 % nitric acid with a heating

block to expedite the reaction. Small quantities of potassium chlorate were added to encourage the process.

In most chimpanzee foods we observed either very few starch grains or none at all, suggesting quantities too negligible to be detected or a complete lack of starch in the plant (Supplementary Table 2). Plants that produced negligible numbers of starches were not analysed for the identification model, because they did not have enough starch grains to build a reference set of 50 starches. We found phytoliths were common in many species, but many morphotypes are poorly studied in morphometric studies and cannot be easily described using the variables we chose for our model (e.g. hair cells, epidermal, cylindroids, plates and tracheid phytoliths). These morphotypes were found in a number of genera in the reference collection plant but only in low numbers.

Plants that had few phytoliths were not included. Furthermore, if microremains were found in parts of a plant that chimpanzees do not eat, the plants were not included (e.g. starch from *Beilschmedia mannii* seed). Thirteen starch- and seven phytolith-producing plants were selected for developing identification criteria. We chose to measure or quantify several variables on 50 microremains per species (Supplementary Data 1 and 2), focusing on variables that past studies have shown to be effective in distinguishing among starches and phytoliths [2,3]. Our variables include max length, max width, area, shape, surface regularity, the number of echinate spines, length of longest cross axis, type, number and length of cracks, number of facets and lamellae (Supplementary Table 8). If abundant starches or phytoliths were recovered, their abundance was analysed in order to assess the expected starch and phytolith contribution to dental calculus (Table 1). Starch content was established by combining previous nutritional content studies[4,5]. For species where this data was not available we assessed starch content per gram dried plant material colourimetrically using an Amyloglucosidase / α-amylase method with a Megazyme Total Assay Kit (AA/AMG 11/01, AOAC Method 996.11, AACC Method 76.13, ICC Standard Method No. 168). Phytolith content was estimated by calculating the total weight of sample left after nitric acid digestion.

4. Identification of microremains by classification

Statistical approaches are increasingly used for the study and classification of microremains[2,6–9]. A variety of approaches have been implemented in past studies such as image analysis[10], linear discrimination[3], and factor regression analysis by principal components[2]. We used random forest-based classification because it is robust, non-parametric and easily accommodates both large number of variables and categorical data. Using this approach, we can easily see the most important variables that drive the differences among the microremain types. The most

important variables in our phytolith model include length and the number of spines (Supplementary Table 8). In the starch random forest model area and length were the most important variables (Supplementary Table 9).

5. Model design and formulae

We predicted that number of microremains should increase with age, and might vary by sex. We tested this using a negative binomial regression, with microremain count as the response, and age and sex as predictors, weighting each observation by the weight of the calculus sample (see detailed methods below). We ran separate tests for phytoliths, unsilicified remains and starches.

The models described in R terminology are as follows:

Microremain type count~ chimpanzee age + chimpanzee sex, weights=calculus sample weight

Expressed as a mathematical formula, this analysis is written as follows:

$$y_i = Negbin(\mu_i, k)$$

$log(\mu_j) = \beta_0 + X_j\beta_j + \varepsilon$

where $\beta_0 = 0$

$$log(\mu_j) = \beta_0 + \sum_{j=1}^{p} [\beta_{11j}\text{chimp\_age}_j + \beta_{12j}\text{chimpanzee sex}_j] + \varepsilon_j$$

where $\beta_0 = 0$

We predicted that more frequently consumed plants should be highly represented in the chimpanzee calculus. To test this, we used an observational random effect Poisson model (Supplementary Text 5). The count of microremains (starches or phytoliths) belonging to a particular genus was our response variable, and the fixed predictors were: (a) minutes spent consuming each genus, and (b) chimpanzee age in months. Sex was included as a control predictor, and both calculus sample weight and successful identification rate of each genus were included as weights. We accounted for the variation in production of microremains in different genera by using microremains content as an offset. We used counts of each genus predicted to be present with the total minutes spent consuming each genus. The chimpanzee individual was included as a random slope term, while year of death, tooth and food type were treated as random intercept terms

The models described in R terminology are as follows:

The observational feeding records model. Key: obs_id=observation id, plant_id= Plant genus, death_year = year that chimpanzee died, mr_content=Prevalence of starch in each plant species, wt= Milligrams in each sample, class_rate=Rate of successful identification in this species.

Count of each plant species~ mins+ age+ sex+ (1|obs_id)+ (1|plant_id)+ (1|tooth)+ (1|chimp_name)+ (1|death_year)+ (0+mins|chimp_name)+ (0+mins|tooth)+ (0+ mins|death_year)+ (0+age|plant_id)+ (0+age|tooth)+ offset(log(mr_content)), weight=class_rate+ calculus samples weight

In mathematical notation, the models are written as follows:

$$
\begin{aligned}
log_e(\lambda) = -n\lambda + log_e(\lambda)\sum_{j=1}^{p}[\,\beta_{11j}\mathrm{mins}_j + \beta_{12j}\mathrm{age}_j + \beta_{13j}\mathrm{sex}_j) + \beta_{21j}{+}u_{11j})\mathrm{tooth}_j \\
+ \left(\beta_{22j}{+}u_{12j}\right)\mathrm{death\_year}_j + \left(\beta_{23j}{+}u_{13j}\right)\mathrm{plant\_id}_j + \left(\beta_{24j}{+}u_{14j}\right)\mathrm{age}_j \\
- \sum_{j=1}^{p}\ln[\beta_{11j}\mathrm{mins}_j + \beta_{12j}\mathrm{age}_j + \beta_{13j}\mathrm{sex}_j) + \beta_{21j}{+}u_{11j})\mathrm{tooth}_j \\
+ \left(\beta_{22j}{+}u_{12j}\right)\mathrm{death\_year}_j + \left(\beta_{23j}{+}u_{13j}\right)\mathrm{plant\_id}_j + \left(\beta_{24j}{+}u_{14j}\right)\mathrm{age}_j]\,! \\
+ u_{01} + u_{02} + u_{03} + u_{04} + u_{05} + \varepsilon_j
\end{aligned}
$$

References

1. Perry, G. H. *et al.* Diet and the evolution of human amylase gene copy number variation. *Nat Genet* **39,** 1256–1260 (2007).

2. Fenwick, R. S. H., Lentfer, C. J. & Weisler, M. I. Palm reading: A pilot study to discriminate phytoliths of four Arecaceae (Palmae) taxa. *J. Archaeol. Sci.* **38,** 2190–2199 (2011).

3. Torrence, R., Wright, R. & Conway, R. Identification of starch granules using image analysis and multivariate techniques. *J. Archaeol. Sci.* **31,** 519–532 (2004).

4. N'guessan, A. K. Aspects quantitatifs et qualitatifs du régime alimentaire des chimpanzés au parc national de Taï, Côte d'Ivoire. (Universite de Cocody, 2012).

5. Oyebade, T. Some aspects of developmental physiology of the Nigerian kola (Cola nitida) fruit. *Econ. Bot.* **27,** 417–422 (1973).

6. Wilson, J. *et al.* Automated classification of starch granules using supervised pattern recognition of morphological properties. *J. Archaeol. Sci.* **37,** 594–604 (2010).

7. Zhang, J., Lu, H. & Huang, L. Calciphytoliths (calcium oxalate crystals) analysis for the identification of decayed tea plants (Camellia sinensis L.). *Sci. Rep.* **4,** 6703 (2014).

8. Coster, A. C. F. & Field, J. H. What starch grain is that? – A geometric morphometric approach to determining plant species origin. *J. Archaeol. Sci.* **58,** 9–25 (2015).

9. Saul, H. *et al.* A systematic approach to the recovery and identification of starches from carbonised deposits on ceramic vessels. *J. Archaeol. Sci.* **39,** 3483–3492 (2012).

10. Colliot, G., C, A. P. & Bonnet, N. in *The State-of-the-Art of Phytoliths in Soils and Plants* 275 (1997).

Supplementary Tables

Supplementary Table 1: Details of the random forest model used to predict genus of origin for the phytolith-producing taxa. Identification rate = rate of successful identification per genus.

Number of variables tried at each split (mtry)						15
Tune length						3
Tree number						500
Out of bag estimate of error rate						25.75%
Confusion matrix						
	Aframomum	*Ancistrophyllum*	*Elaeis*	*Eremospatha*	*Sarcophrynium*	*Identification rate*
Aframomum	39	3	1	5	2	0.78
Ancistrophyllum	3	32	3	12	0	0.64
Elaeis	2	3	40	5	0	0.8
Eremospatha	5	11	1	33	0	0.66
Sarcophrynium	2	0	1	0	47	0.94

Supplementary Table 2: Details of the random forest model used to predict genus of origin for the starch-producing taxa. Identification rate=rate of successful identification per genus.

Number of variables tried at each split (mtry)													14
Tune length													3
Tree number													500
Out of bag estimate of error rate													32.77%
Confusion matrix													
	Aframomum	*Calpocalyx*	*Cola*	*Coula*	*Eremospatha*	*Gilbertiodendron*	*Napoleona*	*Panda*	*Piper*	*Sacoglottis*	*Sarcophrynium*	*Treculia*	*Xylia*	*Identification rate*
Aframomum	45	1	0	0	0	0	0	0	2	0	0	1	1	0.9
Calpocalyx	0	40	0	0	7	0	0	2	0	0	0	0	1	0.8
Cola	0	0	26	0	0	3	0	2	0	5	0	11	3	0.52
Coula	0	0	0	44	3	0	0	0	0	2	0	0	1	0.88
Eremospatha	0	10	0	0	31	0	0	7	0	0	0	0	2	0.62
Gilbertiodendron	0	0	4	0	0	38	1	0	0	7	0	0	0	0.76
Napoleona	0	2	0	0	1	1	18	7	0	2	0	8	11	0.36
Panda	0	3	1	0	6	0	11	11	0	0	0	6	12	0.22
Piper	2	0	0	0	0	0	0	0	47	1	0	0	0	0.94

Sacoglottis	0	0	0	0	0	6	0	0	0	43	0	0	1	0.86
Sarcophrynium	0	0	0	0	0	2	0	0	1	0	47	0	0	0.94
Treculia	0	0	7	0	0	2	4	6	0	1	0	26	4	0.52
Xylia	0	3	0	0	6	0	7	7	0	3	0	3	21	0.42

Supplementary Table 3: Table of total recovered plant microremains, both in the full sample and per milligram of calculus with cause of death of the sampled chimpanzees, condition of their dental calculus and skeleton treatment a) Buried for unknown duration, cleaned and dried (1984-1994, 1996-2004 b) Necropsy, burial for 1 year, possible boiling and dried (1994-1996) and c) Necropsy, burial for 1 year, disinfection with chlorine, 10% formalin and dried (2004-onwards).

Name	Phy	Phy/mg	Starch	Starch/ mg	Unsilicified/ mg	Unsilicified remains	Cause of death	Calculus condition	Skeleton treatment
Ophelia	0	0	1	40	0	0	Pneumonia	White	C
Leonardo	0	0	0	0	0	0	Starvation	White/grey	A
Bambou	0	0	0	0	1	7.41	Tree fall	White	A
Piment	0	0	0	0	0	0	Ebola	White	B
Oreste	40	74.63	4	7.46	1	1.87	Pneumonia	Grey	C
Hector	24	34.83	2	2.9	6	8.71	Anthrax	Orange	A
Noah	47	52.51	2	2.23	32	35.75	Unknown	Brownish	A
Lefkas	19	31.93	11	18.49	13	21.85	Pneumonia	White	A
Tina	29	21.21	8	5.85	6	4.39	Leopard	Brownish	A
Dorry	159	214.29	5	6.74	4	5.39	Unknown	White	A
Zerlina	147	167.43	0	0	9	10.25	Ebola?	Moderate	B
Clyde	27	23.87	4	3.54	3	2.65	Poacher	White	A
Agathe	94	15.47	13	2.14	22	3.62	Ebola?	Brown/creamy	A
Bijou	87	17.26	10	1.98	22	4.36	Unknown disease	Brownish	A
Leo	126	116.13	5	4.61	9	8.29	Unknown	Brownish	A
Castor	65	9.31	25	3.58	6	0.86	Pneumonia	White	A
Fanny	109	27.84	54	13.79	11	2.81	Ebola?	White brown	B
Kendo	233	235.59	0	0	25	25.28	Ebola?	Grey	B
Venus	96	59.26	16	9.88	2	1.23	Unknown	Brownish	C
Goma	98	7.42	181	13.7	17	1.29	Anthrax	White	A
Rubra	120	17.78	10	1.48	30	4.44	Anthrax?	Mixed/white	C
Ondine	26	17	0	0	10	6.54	Ebola?	Brown/ green	A
Mkubwa	11	33.95	0	0	1	3.09	Unknown	Whitish green	A
Brutus	161	49.6	5	1.54	25	7.7	Unknown	Brownish	a

Supplementary Table 4: Summary counts of identified genera in Taï Chimpanzee calculus samples.

	**Phytolith**		**Starch**	
**Name**	**Genera count**	**% of total genera**	**Genera count**	**% of total genera**
Ophelia	0	0	0	0
Leonardo	0	0	0	0
Bambou	0	0	0	0
Piment	0	0	0	0
Oreste	5	100	2	15.38
Hector	3	60	2	15.38
Noah	5	100	0	0.00
Lefkas	2	40	4	30.77
Tina	3	60	2	15.38
Dorry	4	80	3	23.08
Zerlina	4	80	0	0
Clyde	3	60	3	23.08
Agathe	4	80	4	30.77
Bijou	5	100	5	38.46
Leo	4	80	2	15.38
Castor	5	100	3	23.08
Fanny	4	80	10	76.92
Kendo	5	100	0	0.00
Venus	4	80	5	38.46
Goma	5	100	9	69.23
Rubra	5	100	5	38.46
Ondine	3	60	0	0.00
Mkubwa	2	40	0	0.00
Brutus	5	100	3	23.08

Supplementary Table 5: Summary of coefficients of our statistical models.

**Model**	**Term**	**Estimate**	**Std. Err.**	**Z value**	**P**
Tests of effect of age and sex on microremain numbers					
Phytolith Negative binomial	Intercept	3.969	0.160	24.790	1.1398e-135
	Age	0.002	0.0005	3.833	1.2616e-04
	Sex	-0.027	0.157	-0.170	8.6469e-01
Starch Negative binomial	Intercept	3.009	0.426	7.052	1.7575e-12
	Age	0.003	0.001	2.661	7.7805e-03
	Sex	-2.569	0.437	-5.873	4.2665e-09
Unsilicified remains	Intercept	2.210	0.202	10.904	1.0978e-27

Negative binomial	Age	0.001	0.0006	3.093	1.9775e-03
	Sex	-0.048	0.199	-0.245	8.0594e-01
Tests of effect of consumption frequency on microremain numbers					
Phytolith poisson model	Intercept	-0.231	0.876	-0.263	0.791
	z.min	1.707	0.680	2.509	0.0120
	z.age	3.612	2.075	1.740	0.081
	sex	-0.801	0.934	-0.858	0.390
Starch logistic regression model	Intercept	-14.2189228	0.8709593	-6.325589	6.4911e-60
	z.min	0.5912703	0.5056228	1.169390	2.4224e-01
	z.age	0.4893117	0.4425319	1.105709	2.6885e-01
	sex	-1.2664400	0.9964118	-1.271001	2.0372e-01

Supplementary Table 6: Complete inventory of plants and fungus analysed in reference collection. x=no microremain found. o=microremains found and used for identification model. 1=found but not used in classification model due to their complex morphology, 2=found but not included as they are very rare, 3=found but only in parts that are not eaten. Prep=preparation. d=dried, fn=frozen and fh=fresh.

		Leaf	Fruit pulp	Seed	Stem	Pith	Shell	Flower	USO	Bark	Leaf	Fruit pulp	Seed	Stem	Pith	Shell	Flower	USO	Bark	Prep
**Plants**																				
Genus	Species	Starch									Phytoliths									
Aframomum	*exscapum* (Sims) Hepper	x		x																d
Aframomum	*cereum* (Hook.f.) K.Schum.										x		x							d
Afzelia	*bella* Harms										1									d
Agaricus	*bispourus* (J.E.Lange) Emil J. Imbach				x															d
Anchomanes	*difformis* (Bl.) Engl.													x						fn
Antiaris	*toxicaria subsp. welwitschii* (Engl.) C.C.Berg		x	2																d
Auricularia	*auricula-judae.* (Bull.) J.Schröt.				x									x						d
Beilschmiedia	*mannii* (Meisn.) Benth. & Hook.f.			2																d
Bombax	*buonopozense* P.Beauv.			x																d
Bombax	*ceiba* L.	x									2									fh
Calpocalyx	*Sp.*		o																	d
Calpocalyx	*aubrevillei* Pellegr.	x									x									d
Canarium	*schweinfurtii* Engl.		x	x																fn
Castanola	*paradoxa* (Gilg)											x	x							d

	Schellenb.																			
Chrysophyllum	*taiense* Aubrév. & Pellegr.	x	x	x							x	x	x							d
Cola	*nitida* (Vent) Schott & Endl.	x	x	x							1	x	x							d, fh
Cola	*heterophylla* (P Beauv.) Schott. & Endl.	x	x	x							1	x	x							d
Cola	*laterita* K Schum.											x	x							d
Cordia	*platythyrsa* Baker		x	x								x	x							d
Coula	*edulis* Baill.	x		x			x				1		x			1				d
Dacryodes	*klainaea* (Pierre) H.J.Lam		x									x								fn
Desplatsia	*chrysochlamys* (Mildbr. & Burret) Mildbr. & Burret	x									x									d
Detarium	*senegalense* J.F.Gmel.											x	x							d
Dialium	*aubrevillei* Pellegr.	x	x								x	x								d
Dialium	*dinklagei Harms*		x	x																d
Dichapetalum	*heudelotii* (Planch.) Baill.	x									x									d
Dioscorea	*burkilliana* J.Miège																	x		d
Diospyros	*chevalieri* De Wild.											x								d
Diospyros	*manii Hiern*		x								x	1								d
Diospyros	*sanza minika* A Chev.											x								d
Diospyros	*soubreana* F.White										x									d
Drypetes	*aubrevillei* Léandri						x						x			x				d
Duboscia	*viridifolia* (K.Schum.) Mildbr.		x																	d
Duguetia	*staudtii* (Engl. & Diels) Chatrou		3	3																d
Elaeis	*guineenis* Jacq.	x	x								o	o		o						d, fh
Entandrophragma	*angolense* (Welw.) C. DC.		x	x																d
Eremospatha	*macrocarpa* H.Wendl.					o									o					d
Erythrophleum	*ivorensis* A.Chev												x							fn
Ficus	*barteri* Sprague										1									d
Ficus	*elastica* Roxb.		x								1									fh
Ficus	*elasticoides* De Wild		x																	d
Ficus	*lutea* Vahl		x																	d
Ficus	*polita* Vahl										1									d
Gilbertiodendron	*splendidum* (Hutch. & Diels) J. Léonard		o	o								x	x							d
Glyphaea	*brevis* (Spreng.) Monach.	x									3									d

Grewia	*biloba* (Bunge.)Hand. Mazz.		x	x							x	x								d
Grewia	*malacocarpa* Mast.		x	x																d
Guibourtia	*tessmannii* (Harms) J.Léonard																x			d
Halopegia	*azurea* (K.Schum.) K.Schum.					x		x			x	x			x		x			d
Harungana	*madagascariensis* Lam. ex Poir.		x	x																fn
Heisteria	*parvifolia* Sm.			x																d
Hexalobus	*crispiflorus* A.Rich			x																fn
Hypselodelphys	*violacea* (Ridl.) Milne-Redh					x									1					d
Irvingia	*gabonensis* (Aubry-Lecomte ex O'Rorke) Baill.		x	x																d
Irvingia	*grandifolia* (Engl.) Engl.		x																	d
Keayodendron	*bridelioides* (Gilg & Mildbr. ex Hutch. & Dalziel) Leandri		x																	d
Klainedoxa	*gabonensis* Pierre		3																	fn, d
Laccosperma	*secundiflorum* (P.Beauv.) Kuntze					x									x					d
Laccosperma	*opacum* Drude					x									x					d
Landolphia	*dulcis* (Sabine ex G.Don) Pichon		x									x							x	fn
Magnistipula	*butayei* DeWild		x																	d
Mammea	*africana* Sabine		x									x								d
Manilkara	*obovata* (Sabine & G.Don) J.H.Hemsl.		x	x																fn
Manniophyton	*fulvum* Müll.Arg.	x																		d
Memecylon	Sp.		x																	fn
Musanga	Sp.		x									1	1							d
Myrianthus	Sp.									x										fn
Myrianthus	*arboreus* P.Beauv.		x																	fn
Napoleona	*leonensis* Hutch. & Dalz.			o																d
Napoleonaea	*vogelii* Hook. & Planch	x									x		x							fh
Nauclea	*diderrichii* (De Wild. & T.Durand) Merr.ill		x																	d
Nauclea	*xanthoxylon* (A.Chev.) Aubrév.		x									x								d
Pachira	*cubensis* (A.Robyns) Fern.Alonso	x																		fh
Palisota	*barteri* Hook.f.		2	2								x	x		x					d
Palisota	*bracteosa* C.B.Clarke		x	x																d

Palisota	*hirsuta* (Thunb.) K.Schum.														x					d
Panda	*oleosa* Pierre	x		o							x		x							d
Parinari	*excelsea* Sabine	x	x								1	x								fn
Parkia	*bicolor* A.Chev.		x									x								fn
Pentaclethra	*macrophylla* Benth							x												d
Pentaclethra	*macrophylla* Benth							x									x			d
Pentadesma	*butyracea* Sabine		x																	fn, d
Piper	*betle* L.	x						x			1						1			fh
Piper	*guineense* Schumach. & Thonn.		o	o																d
Piper	*longum* L.		x	x																d
Piper	*arboreum* Aubl.	x									1									fh
Piper	*ornatum* N.E.Br.	x																		fh
Pouteria	*pierrei* (A.Chev.) Baehni		x	x								x	x							d
Pseudospondias	Sp.		x	x																fn
Pseudospondias	*microcarpa* Engl		x	x																d
Psychotria	*bacteriophila* Valeton		x	x																d
Pycnanthus	*angolensis* (Welw.) Warb.		x																	d
Raphia	*sudanica* A.Chev.														x					d
Rhodognaphalon	*brevicuspe* (Sprague) Roberty		x	x																d
Rudgea	*ciliata* (Ruiz & Pav.) Spreng.	x	x	x							x									d
Sacoglottis	*gabonensis* (Baill.) Urb.	x	o								1	1								d
Sarcocephalus	*pobeguinii* Hua ex Pobég		x																	d
Sarcophrynium	*prionogonium* (K.Schum.) K.Schum.		o	o								o	x							d
Scottellia	*coriacea* A.Chev. & al.		x																	d
Scytopetalum	*tieghemii* Hutch. & Dalziel	x																		d
Strombosia	*glaucescens* Engl.											x								d
Strychnos	*aculeata* Soler.	x	x								x	x	x							d
Syzygium	*guineensis* (Willd.) DC.			3																fh
Syzygium	*paniculatum* Gaertn.	x	2	2				x			1		1							fh
Tamitia	*utilis*										x									d
Treculia	*africana* Decne. ex Trécul	x	x								x	2	x							d
Trichophyton	*Sp.*							x												d
Trichoscypha	*arborea* (A.Chev.) A.Chev.		x	3																d

Triclisia	*macrophylla* (Baill.) Diels		x									1								d
Tristemma	*hirtum* P.Beauv.		x																	d
Uapaca	*corbisieri* DeWild.	x	x								x	x								d
Uapaca	*guineensis* Müll.Arg.											x								fn
Uvariastrum	*pierreanum* Engl. & Diels		x	x								1								d
Vitex	*doniana* Sweet		x	x																fn
Xylia	*evansii* Hutch.	x		o							1									d
Xylopia	*quintas* Pierre ex Engl. & Diels		x	x																d
Xylopia	*villosa* Chipp									x										d
Zanha	*golungensis* Hiern		x	x																d
**Fungus**																				
Agaricus	*bispourus* (J.E.Lange) Emil J. Imbach				x															d
Auricularia	*auricula-judae.* (Bull.) J.Schröt.				x									x						d

Supplementary Table 7: Summary of microremain variables used for identification model.

**Variables Shared**	**Description**	Metric
Length	Maximum diameter (µm), measured from spine tip to spine tip	Numeric (µm)
Width	Maximum diameter (µm) perpendicular to the maximum diameter	Numeric (µm)
LW Ratio	Length to width ratio	Numeric (µm)
Area	Total observable area in a 2D plane	Numeric (μm^2)
Shape	Ovoid, elongate ovoid, pyriform, oblate conovoid, elongate conovoid, hemispherical, triangular, quadrangular, polygon, polygon concave-convex, angularpoint, angulate elongate, ovoid concave-convex, prolate concave	16 descriptors
**Starch specific**		
Facets	Total number of maximum observable facets	Counts
Lam	Lamellae presence and distinctness	0-3 scale
Dist	Distance of longest arm of cross observed on cross-polarised light	Numeric
Striaelen	Average length of radial striae/cracks visible on the starch	Numeric
Striaeno	Number of radial striae/cracks visible on the starch	Counts
Type	simple, semi-compound or compound classification	3 descriptors
**Phytolith specific**		
Irregul	Measure of phytolith surface irregularity	0-4 scale
Spinelen	Estimated mean spine length: the mean length of spines approximately parallel with the viewing plane	Numeric (µm)
Spineno	Number of spines visible in entirety in the viewing field. Spines were counted value if their base was not obscured by the phytolith.	Numeric
Conjoined	Score of phytolith attachment to other phytoliths	1-2 scale

Supplementary Table 8: Variable importance in phytolith random forest.

Variable	Importance
Length	100.00
Spine number	75.301
Spine ang	74.109
LW Ratio	43.996
Spine length	42.854
Area	29.581
Width	22.056
Irregul	10.236
Spherical	6.667
Angularpoint	6.575
Polygon	4.590
Ovoid	1.663
Prolate	1.620
Triangular	1.447
Elongate	0.440
Quadrangular	0.228
Facets	0.184
Conjoined	0.106
Prolate concave-convex	0.043
Polygon concave	0.042

Supplementary Table 9: Variable importance in starch random forest.

Variable	Importance
Area	100
Length	75.8434
Width	67.5876
Dist	61.1718
Facets	60.4963
LW Ratio	56.2298
Type	55.9587
Lam	35.8372
Spherical	31.833
Prolate	8.2554
Ovoid	7.157
Polygon	5.8693
Hemispherical	4.9279
Oblate conovoid	4.6926
Striaelen	2.4395
Elongate ovoid	2.4011
Striae no	2.1051
Triangular	1.8956
Quadrangular	0.9141
Pyriform	0.4986

Supplementary Figures

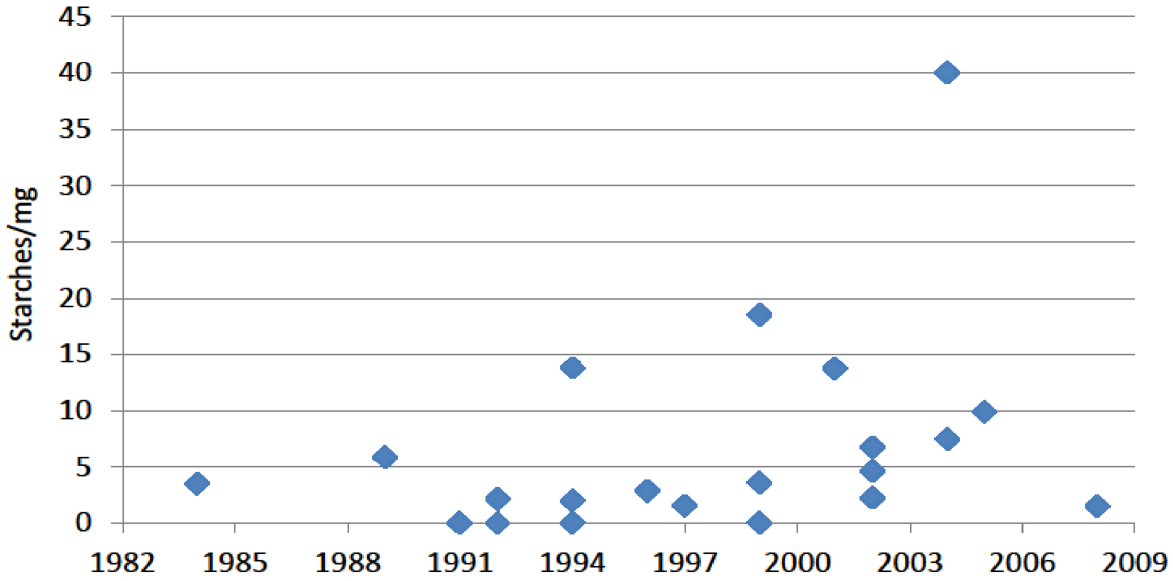

Supplementary Figure 1: Scatter plot of starches per mg in each chimpanzee calculus samples and year of chimpanzee death. Starches /mg incudes the possible starch microremain category. Treatment of the skeletal remains and year of chimpanzee death does not predict variation of starches per mg.

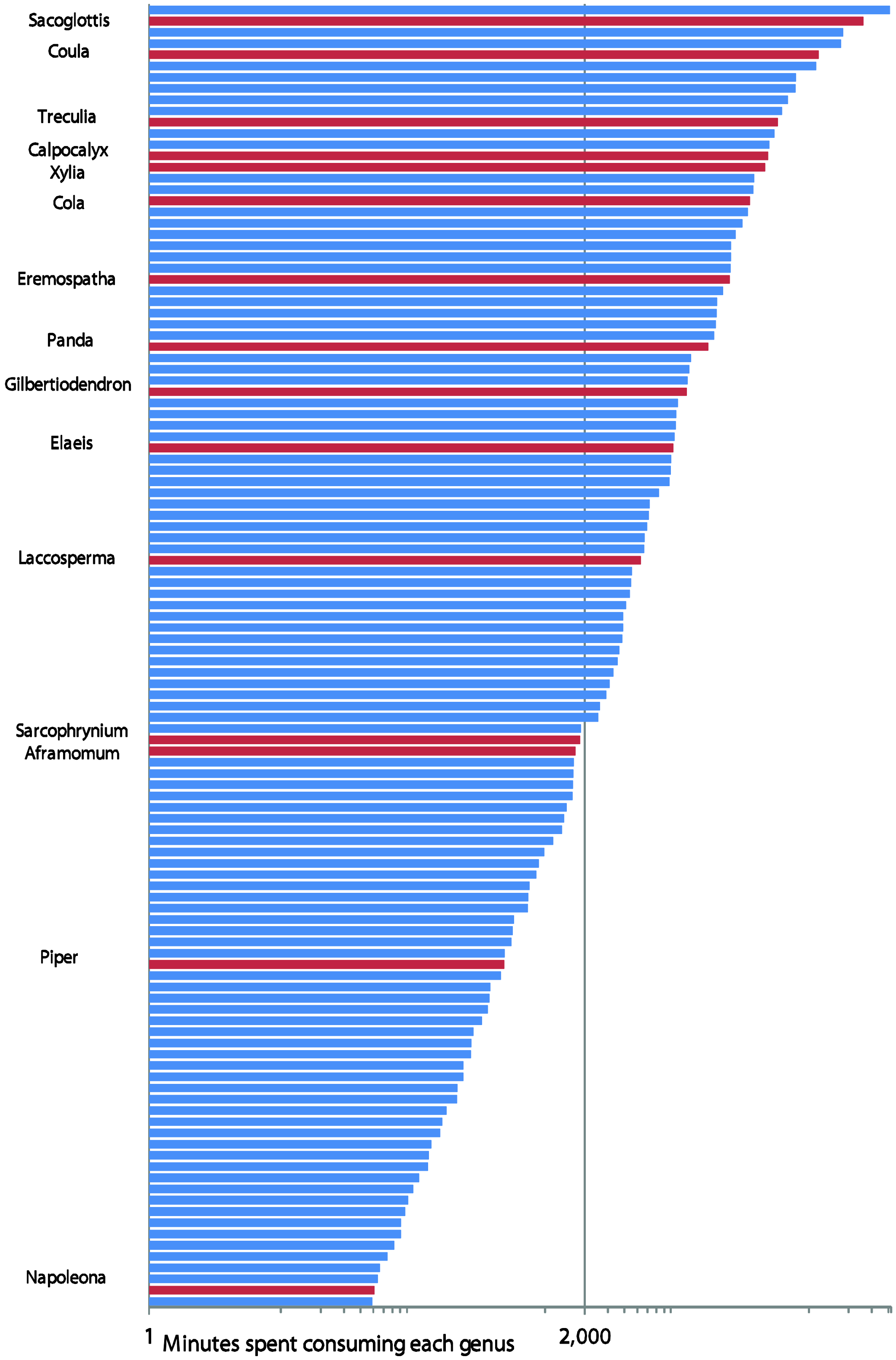

Supplementary Figure 2: Bar chart of chimpanzee plant foods, ranked by the number of minutes each was consumed. Plants in random forest model are in red and those that are not

are in blue. Chart omits foods eaten for <40 minutes. Our sample includes plants that are frequently consumed (e.g. *Sacoglottis* and *Coula*) as well as those less often eaten (e.g. *Piper* and *Napoleona*).
